# Dental radiography image enhancement for treatment evaluation through digital image processing

**DOI:** 10.4317/jced.54607

**Published:** 2018-07-01

**Authors:** Hanifah Rahmi-Fajrin, Sartika Puspita, Slamet Riyadi, Erma Sofiani

**Affiliations:** 1 S.T., M. Eng, Dept. Electromedical Engineering. Vocational Program, Universitas Muhammadiyah Yogyakarta, Indonesia; 2 DDS, M.D.Sc, Dept. Oral Biology. School of Dentistry, Faculty of Medical and Health Sciences, Universitas Muhammadiyah Yogyakarta, Indonesia; 3 S.T., MSc, PhD, Dept. Informatic Engineering. Faculty of Engineering, Universitas Muhammadiyah Yogyakarta, Indonesia; 4 DDS, Sp.KG, Dept. Endodontic and Conservative Dentistry. School of Dentistry, Faculty of Medical and Health Sciences, Universitas Muhammadiyah Yogyakarta, Indonesia

## Abstract

**Background:**

Evaluation of dental treatment is performed by observing dental periapical radiography to obtain information of filling’s condition, pulp tissue, remain dentin thickness, periodontal ligament, and lamina dura. Nevertheless, the radiographic image used often has low quality due to the level of x-ray radiation made low purposely in order to prevent health problem and limited tools capability. This low quality of the radiographic image, for examples, low image contrast, less brightness, and noise existence cause periapical radiography evaluation hard to be performed. This study aims to improve dental radiographic image quality for assisting pulp capping treatment evaluation.

**Material and Methods:**

The research methodology consists of three main stages, i.e. data collection, image enhancement method production, and result validation. Radiographic image data collection in The Dental Hospital UMY. Image enhancement method has been conducted by comparing several methods: contourlet transform (CT), wavelet transform, contrast stretching (CS), and contrast limited adaptive histogram equalization (CLAHE) to reduce noise, to optimize image contrast, and to enhance image brightness.

**Results:**

The result of this study is according to mean square error (MSE) and peak signal to noise ratio (PSNR) statistics evaluation, it obtains that the highest scores of MSE and PSNR in row gained from CT method totaled 5.441453 and 40.53652, followed by CLAHE method with the scores are 10.66326 and 38.00736, CS method whose scores are 12.39881 and 39.18518, and the last is wavelet method with the scores are 15.41569 and 36.25343.

**Conclusions:**

Nonetheless, MSE and PSNR scores are not enough merely to give a recommendation of any suitable methods for improving contrast, therefore, it needs another success parameter coming from the dentist.

** Key words:**Dental radiography, image enhancement, digital image processing.

## Introduction

Pulp capping treatment is indicated for reversible pulpitis. The initial success of the treatment is seen by the dentist after one-week application of pulp capping material based on subjective and objective examination. Subjective examination based on anamnesis, the patient is not feeling pain anymore and no complaints during the treatment. The objective examination by sondasi, percussion, and palpation with the result in normal limit. A radiographic examination was performed by the dentist as a follow-up investigation after one week of pulp capping treatment which was to see the condition of pulp tissue, periodontal ligament, lamina dura, the lining and pulp capping material were in hermetic condition. Furthermore, after more than 1 month of the treatment, the dentist will control the pulp capping treatment condition by investigation of the radiograph to see the tertiary dentin formation. However, to determine the thickness of tertiary dentin is still a difficulty for most dentists. So, it is necessary to make dental image enhancement (1).

The radiography in dental observation and treatment evaluation has been used commonly in many hospitals and clinics. Radiographic images are obtained from X-ray radiation passing through mouth structure with different levels based on organ density so that it produces various image greyscale level (2). X-ray radiation intensity is adjusted as small as possible, about 0.150 mSv according to American Dental Association, in order not to damage dental and organ tissue in the mouth (3-5). The effect of radiation should be adjusted as small as possible. Radiographic equipment will produce radiographic images with less contrast resulting teeth object, especially the edge parts, getting blurred (6-8). Besides the aspect of the radiation generating less contrast image, another one influencing Rontgen image quality is the limitation of the equipment’s capability itself so that the images production containing much noise. Its existence is disturbing enough due to causing teeth object covered by unnecessary information. Another problem occurred in the radiographic image is incorrect brightness level, so the teeth object does not display sharply (9,10). The parts of detailed teeth are invisible making the doctors difficult to have images observation. In short, radiographic images producing noise, fewer contrast images, and incorrect brightness level causing the doctors hard to evaluate dental periapical, particularly for clerkship in dental hospital (11,12).

In this image processing discipline, various methods for improving contrast and brightness as well as reducing noise have been developed by researchers. Those methods can be categorized into two approaches, i.e. spatial and frequency domain. Spatial domain approach is very famous because the method is implemented directly in every image pixel, by processing every pixel value itself and also considering neighbor pixels (13). One of the methods applying this approach is histogram equalization (HE). It is popular due to being accorded with human logic when seeing an object that is intensity distribution of pixel values globally and locally (14).

The use of HE method to enhance radiographical image has already limited to deal with one of the three problems as stated before that are less contrast, incorrect brightness, and noise existence (15). Therefore, it needs a new method to be developed that is capable to enhance radiographical image quality by dealing with all of the problems, thus the images are able to provide more significant information for doctors and to facilitate radiographic image evaluation (16). By comparing contrast enhancement methods to attain the best method recommendation, hopefully, it can assist dentists to evaluate treatment.

## Material and Methods

**Figure 1:**
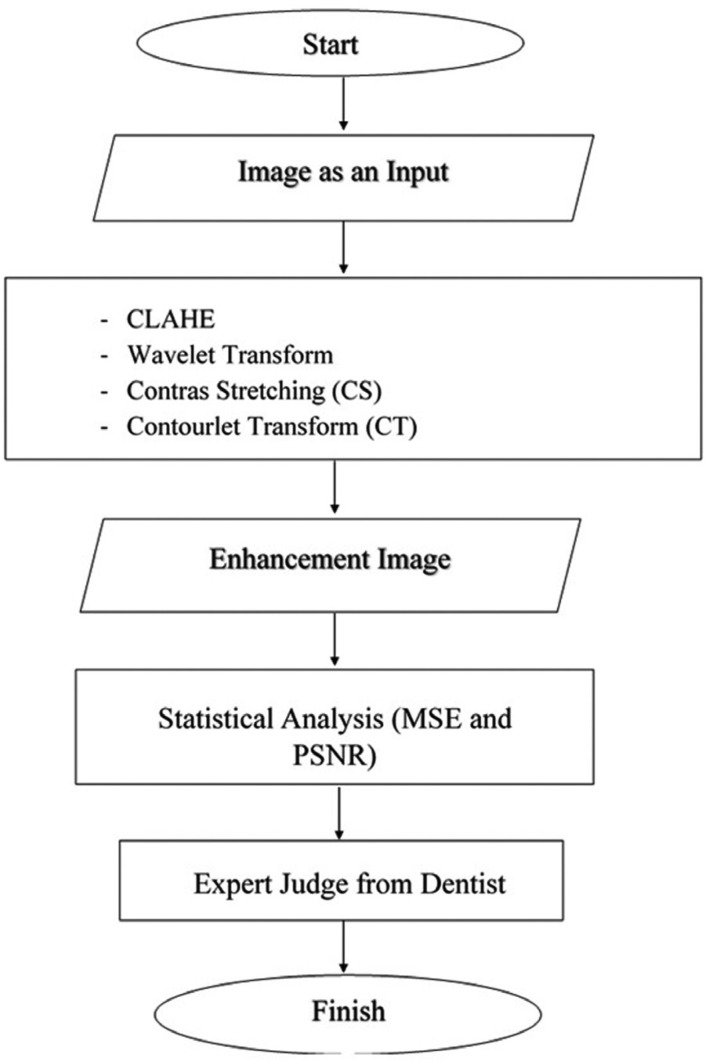
Flowchart of research.

As seen on Figure 1, the material and methods of this research as follow:

a. Compiling radiographic image enhancement method

The radiographic digital image is a red green blue image (RGB), although it seems to be grayscale image visually. RGB images should be turned into grayscale images to make processing easily and rapidly performed (6,7,17). Then, the images would be processed by applying wavelet, contourlet transforms, contrast stretching, and CLAHE methods with the purpose that was to reducing image noise and optimizing contrast by still maintaining image brightness (6,8,17). From several methods used, it would select the best method in the dental radiographic images quality enhancement process. This stage conducted in the laboratory or office using the main equipment was computer and software MATLAB (13). The output of this stage was an image enhancement method with the success indicator of the better image quality enhancement that can be found in the validation stage (18,19).

b. Result validation

This stage aims to investigate whether the method produced is successful to enhance image quality and to assist pulp capping treatment evaluation. It conducted by applying two methods as follows:

1) Statistical analysis: It is used to investigate method effectivity in image quality enhancement capability generally by using MSE and PSNR parameters (20).

2) Expert judge: It is a validation involving specialist doctor as a judge towards a validated object. Evaluation results from a general dentist and dental conservative specialist were used to be the ground truth that would be used to examine image processing method performance. The way of validation performed was dentist and dental conservation specialist conducted visual observation on before and after images of implementation of the correction methods and determining image quality level by operational definition (OD) indicators, for instances very easy (VE), easy (E), Difficult (D), and very difficult (VD). The before and after images of enhancement method implementation were given randomly to increase visual observation objectivity (21-24).

There are four parameters of radiographic image processing result examination as follows (21,25-27):

P1 = Density of the restoration (D)

P2 = Pulp Tissue (PT)

P3 = Remain Dentin Thickness (RDT)

P4 = Lamina Dura (LD).

Operational Definition (OD) for image process results consisted of four levels that are (21) :

1. VD (Very Difficult)

a. Dental anatomy was unable to be observed

b. Contrast and brightness were too extreme

c. Limitation of radiopaque and radiolucent was blurred 

2. Difficult (D)

a. Dental anatomy was able to be observed

b. Contrast and brightness were flat

c. Limitation of half section of radiopaque and radiolucent

3. Easy (E)

a. Dental anatomy was able to be observed wholly easily

b. Contrast and brightness were good (both radiopaque and radiolucent were good)

c. Limitation of radiopaque and radiolucent was obvious by careful observation

4. Very Easy (VE)

a. Dental anatomy could be observed very easily obviously

b. Contrast and brightness were excellent (anatomical illustration observed was crystal clear without careful observation)

In every image evaluation, the doctor wrote down ease and certainty levels of evaluation result had been performed (28,29). The evaluation using analysis method conducted by experts, general dentist and dentist specialize in conservative dentistry was treated to 10 patients that each of patient whose 3 images, such as first visit (indication) image, second visit image (temporary restoration), and third visit image (permanent restoration) (30). Every original image had been processed by four methods, thus it totaled 150 images observed. Image quality enhancement methods consisted of CS, CLAHE, CT, and Wavelet Transforms. Gaining the more objective evaluation of the methods used, each method was stated by initial, for examples, A for CS, B for CLAHE, C for CT, D for wavelet transform, and E is for an original image. The experts did not know those initials, hence could give evaluation scores freely.

The process of image quality enhancement in this research conducted by using several methods, for instances, CLAHE, wavelet transform, CS, and CT. For each method applied, it would be evaluated by two methods. First, it compared between original images and processed images (enhanced quality) so that it gained the statistical score. MSE and PSNR were used as statistical scores. From those two scores, we would see how far each method was able to maintain information on images. Second, it was obtained from expert’s analysis (dentist).

## Results

**Figure 2:**
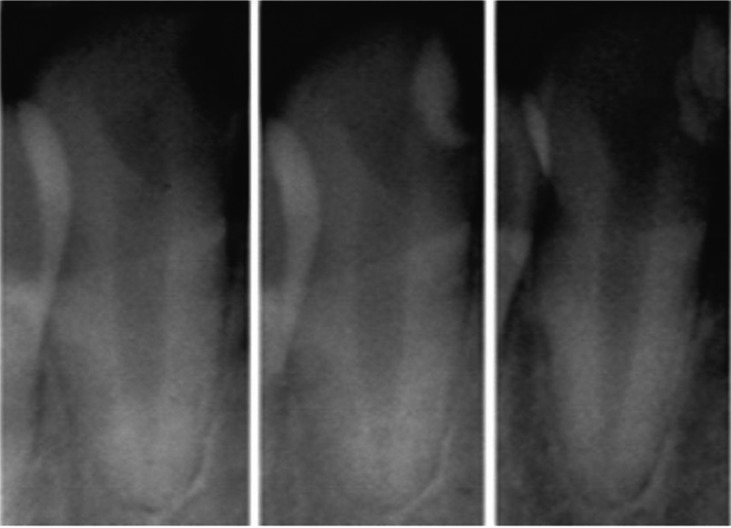
Original images for (a) first, (b) second and (c) third visit to dentist.

**Figure 3:**
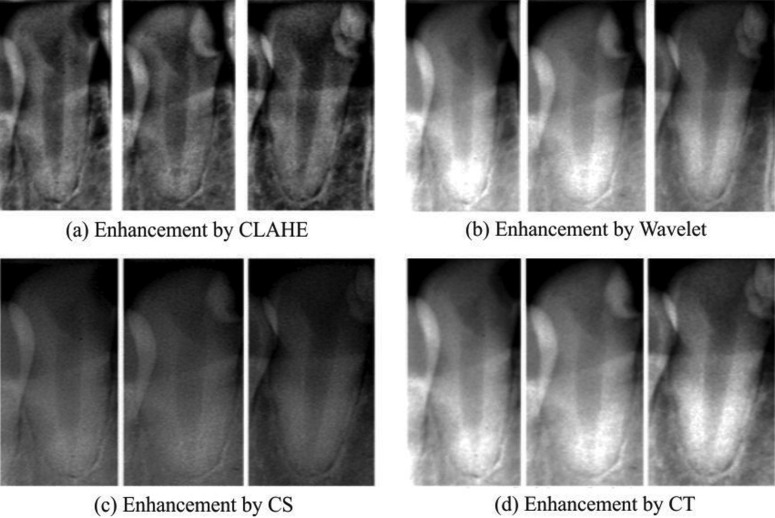
.Image enchanment by (a) CLAHE, (b) walete transfort, (c) contrast streching, (d) contourlet transform methods.

There were 10 patients participating in this study. Each of them did observe three times (indication, 1 week after treatment, and more than one month after treatment) (5,31). The doctor would check or make sure the parts of teeth that would be filled by restoration as the treatment in the first visit. In the second visit, the dentist would fill restoration in the cavities, however the fillings were temporary. In the third, it would make restoration the cavities permanently (31,32). In other words, there were 150 images used in this research. To see the results of dental image quality enhancement, we can look at Figure 2 and Figure 3.

Statistical Analysis (MSE and PSNR) from the experiment results on four methods used, it obtained MSE and PSNR mean scores as we can see in  Table 1.

**Table 1:**
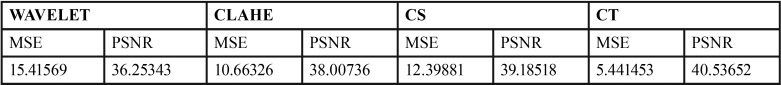
Mean Square Error (MSE) and Peak Signal to Noise Ratio (PSNR) statistics evaluation of Wavelet Transform, CLAHE, Contourlet Transform (CS) and Contrast Stretching (CT) Method.

According to  Table 1, indicated that CT method gained the highest MSE and PSNR mean scores if it compared by the others, that were 5.441453 and 40.53652, followed by CLAHE whose scores of 10.66326 and 38.00736, then CS whose scores of 12.39881 and 39.18518, and the last but not the least was wavelet whose scores of MSE 15.41569 and PSNR 36.25343. Nevertheless, these two scores merely were insufficient for providing the suitable method recommendation used in contrast enhancement because the methods did not count features of the domains where the pixel value change existed, just counted the comparison of square average total among image pixels (20), hence it needed another success parameter, i.e. expert judge analysis (dentist).

According to the  Table 2, for parameter P1 (Filling Density), the best result derived from CLAHE method (B) with Operational Definition (OD) Easy (E) totaled 15 images and OD Very Easy (VE) totaled 13 images. The total of an image in the OD range with an evaluation that was clear and observed easily (E and VE) totaled 28 images. It was followed by CS (A) totaled 22 images for E and VE. On the one hand, both CT (C) and the original image (E) resulted from 22 images. And the last but not the least, wavelet enhancement method (D) produced 19 images.

**Table 2:**
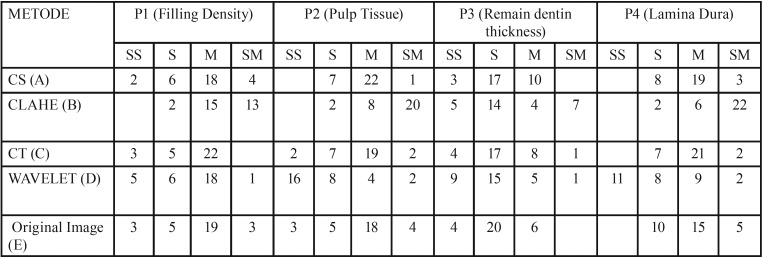
Image Processing Analysis Result Based on The Condition of Filling Density, Pulp Tissue, Remain Dentin Thickness and Lamina Dura.

For parameter P2 (Pulp Tissue), the best score was represented by OD Easy and Very Easy found in CLAHE method (B) whose 28 images. It was continued by CS method (A) totaled 23 images, an original image (E) totaled 22 images, CT (C) totaled 21 images, and then wavelet (D) totaled 6 images.

It was quite hard to observe Parameter P3 (RDT), even its quality had been enhanced by those methods. The best method in this parameter was represented by CLAHE (B) whose 11 images. It was followed in the row by CS method (A) totaled 10 images, CT (C) totaled 9 images, wavelet (D) totaled 6 images, and the last was the original image (E) totaled 6 images.

Finally, the best method for Parameter P4 (Lamina Dura) was CLAHE (B) totaled 28 images, continued by CT method (C) totaled 23 images, CS method (A) 22 images, original images (E) totaled 20 images, and then Wavelet (D) totaled 11 images.

## Discussion

Base on the two types of analysis methods on dental radiography images, both MSE and PSNR statistical methods as well as according to expert’s observation (dentist), it occurred score difference (4), that was based on MSE and PSNR statistical scores, it obtained the best score derived from CT method whose MSE and PSNR scores that were 12.39881 and 39.18518. However, according to dentist’s observation through several parameters required for evaluating filling density that were P1 = Filling Density (FD), P2 = Pulp Tissue (PT), P3 = Remain Dentine Thickness (RDT), and P4 = Lamina Dura (LD), it obtained the best scores for P1 – P4 in CLAHE method.

In this research, the researchers stood on the experts’ observation results because MSE and PSNR scores were inadequate enough to provide the suitable method recommendation used in contrast enhancement. It was because those scores did not count feature of the domain where the pixels score change existed yet did only the difference of square average total among images pixels (20).

## Conclusions

Based on the research has been conducted on the four methods of image quality enhancement, it can be concluded that:

1. CT method gained the highest MSE and PSNR mean scores if it compared by the others, that were 5.441453 and 40.53652 followed by CLAHE whose scores of 10.66326 and 38.00736, then CS whose scores of 12.39881 and 39.18518, and the last but not the least was wavelet whose scores of MSE 15.41569 and PSNR 36.25343.

2. CLAHE method (B) contributed good evaluation in P1-P4 parameters. Pulp Tissue (PT) and Lamina Dura (LD) looked very clear, nevertheless, less clear Filling Density (FD) and Remain Dentine Thickness (RDT) due to declining density became their weaknesses.

3. Wavelet method (D) had no significant diagnostic at all due to too bright. Consequently, its score tended to be lower as well as MSE’s and PSNR’s.

4. CS (A) and CT (C) methods tended to have the similar capability in distinguishing the four parameters given by the experts. MSE and PSNR scores were also about to approach. The images generated by these methods looked too bright in some cases, therefore, it was hard to evaluate filling.
